# Antidepressants and Vertebral and Hip Risk Fracture: An Updated Systematic Review and Meta-Analysis

**DOI:** 10.3390/healthcare10050803

**Published:** 2022-04-26

**Authors:** Renato de Filippis, Michele Mercurio, Giovanna Spina, Pasquale De Fazio, Cristina Segura-Garcia, Filippo Familiari, Giorgio Gasparini, Olimpio Galasso

**Affiliations:** 1 Psychiatry Unit, Department of Health Sciences, University Magna Graecia of Catanzaro, 88100 Catanzaro, Italy; defilippisrenato@gmail.com (R.d.F.); defazio@unicz.it (P.D.F.); 2 Department of Orthopaedic and Trauma Surgery, “Mater Domini” University Hospital, V.le Europa (loc. Germaneto), “Magna Græcia” University, 88100 Catanzaro, Italy; giovanna.spina92@gmail.com (G.S.); filippofamiliari@unicz.it (F.F.); gasparini@unicz.it (G.G.); galasso@unicz.it (O.G.); 3 Psychiatry Unit, Department of Medical and Surgical Sciences, University Magna Graecia of Catanzaro, 88100 Catanzaro, Italy; segura@unicz.it

**Keywords:** adverse drug reaction, antidepressant, selective serotonin reuptake inhibitors (SSRI), bone mineral density, falls, fracture, hip, vertebrae, major depressive disorder (MDD), pharmacovigilance

## Abstract

Although antidepressant drugs appear to play an active role in increasing fracture risk, their weight is still unclear. We conducted a PRISMA compliant systematic review and meta-analysis through PubMed/Scopus/Cochrane libraries and registered with PROSPERO (registration number CRD42021254006) to investigate the relationship between antidepressant drugs categories, including SSRIs, SNRIs, and TCAs, and the risk of hip and vertebral fractures. After screening 3122 items, we finally found 26 papers for qualitative analysis and 11 for quantitative synthesis. A total of 15,209,542 adult and elderly patients were identified, with a mean follow-up of 51 months and a major prevalence of women. We identified results largely for SSRIs, with only a small amount of data for SNRIs, TCAs, and NaSSA. No data were found among the most recent categories of antidepressants, such as vortioxetine and esketamine. All included studies reported hip fractures, while three of them also included vertebral fractures. Overall, we observed a significant effect of SSRIs on fracture risk with a mean effect of 0.98 (95% CI = 0.75–1.20). This meta-analysis reveals that the use of SSRIs increases the risk of fractures. Clinicians’ awareness in antidepressant prescription should optimize their potential while reducing this risk.

## 1. Introduction

Antidepressant drugs are the most prescribed treatment for depression. The prevalence of antidepressant use in older adults ranges from 15% to up to 30%. Antidepressants are also increasingly used for anxiety, neuropathic pain, and insomnia, or for treatment of minor depressive symptoms. However, antidepressants have been shown to be associated with both a decrease of the bone mineral density (BMD) and an increased fracture risk [1]. Sedation, orthostatic hypotension, arrhythmias, hyponatremia, and confusion were suggested as the potential mechanism that causes the increased fracture risk [2]. Fractures, especially of the hip and vertebrae, result in pain, inability to walk, and reduction in the quality of life that carry a high risk of adverse health outcomes, economic implications, and mortality [3,4,5].

Selective serotonin reuptake inhibitors (SSRIs) are the most utilized group of antidepressants, constituting more than 60% of all antidepressants prescribed worldwide [6], and they are considered safer and better tolerated than other types of antidepressants [7]. However, it has been shown that SSRIs could be associated with a higher risk of falls [8]. Moreover, serotonin receptors and the serotonin transporters have been reported in bone, suggesting that antagonizing serotonin reuptake could modulate bone homeostasis. Indeed, inhibition of serotonin transport systems could reduce the stimulating effect of serotonin on the proliferation of precursor cells of osteoblasts, favoring a loss of BMD. Systematic reviews incorporating studies on SSRIs and serotonin-norepinephrine reuptake inhibitors (SNRIs) up to April 2019 found that SSRIs may be associated with an increased fracture risk and recommend physicians to considerer bone health when prescribing this class of drugs [9]. Wu et al. reported that SSRIs may exert an increased risk of fracture independent of depression and BMD [7]. On the contrary, a meta-analysis of four studies concluded that antidepressants including tricyclic antidepressants (TCAs) and SSRIs have no impact on BMD [10] and fracture risk [11].

Currently, the role of antidepressant treatment in hip and vertebral fracture risk remains disputed. New clinical studies reporting outcomes on a larger number of patients, newer class of drug, and indications are emerging.

This systematic review aims to investigate the relationship between antidepressant drugs categories, including SSRIs, SNRIs, and TCAs, and the risk of hip and vertebral fractures.

## 2. Materials and Methods

We conducted a systematic review fitting the methods recommended by the Cochrane Collaboration and reported all the search steps and results in accordance with the most updated preferred reporting items for systematic reviews and meta-analyses (PRISMA) guidelines [12]. The search strategy was previously registered on the PROSPERO database (registration number CRD42021254006).

### 2.1. Search Strategy

We screened PubMed/Medline, Cochrane Database, and Scopus libraries and electronic databases to identify eligible items, with a search restricted to English language only. The search was performed from inception to 1 February 2022 by entering the following search string for the topics of antidepressants and risk fracture, written applying a combination of MeSH terms and keywords as following: (antidepressant OR tricyclic OR SSRI OR SNRI OR “Selective Serotonin Reuptake Inhibitors” OR “Serotonin and Norepinephrine Reuptake Inhibitors” OR Vortioxetine OR Esketamine) AND (“risk fracture” OR fracture OR “bone mineral density” OR BMD OR bone OR osteoporosis OR osteopenia OR “bone loss” OR “bone health” OR “bone metabolism”).

Additional gray literature and internet searches (Google and Google Scholar) were also completed to identify potentially eligible unpublished studies. In addition, hand-searches of previous similar screened systematic reviews and references of included papers were conducted with the goal to find any potential missing suitable studies.

### 2.2. Inclusion Criteria and Study Selection

We included in the final analysis only studies describing any antidepressant intervention prescribed for any disease and psychiatric disorder diagnosed according to the Diagnostic and Statistical Manual (DSM) of mental disorders DSM-IV, DSM IV-TR, DSM-5, or International Classification of Diseases (ICD) ICD-10 or ICD-11 criteria, or through validated scales with cut-off, or clinical records, where the hip and/or vertebral risk of fractures has been assessed or evaluated. Only papers fulfilling the described inclusion/exclusion criteria were deemed eligible for inclusion, according to the population, intervention, control, and outcomes (PICO) methodology [13,14]. We included all clinical trials and observational study designs, except for case reports/series, including retrospective, cross-sectional, or prospective design. Articles presenting narrative and systematic reviews, meta-analysis, umbrella reviews, commentaries/opinion pieces, expert opinions, editorials, letters to the editor, and book chapters were excluded from the analysis but considered for the discussion section. Studies evaluating only the point of view of a patient or caregiver were not deemed eligible for the present review.

Subjects included in the studies could have been either chronically or in the early phase of diseases and could be both with and without a previous personal psychotropic drug history. We excluded studies evaluating patients affected by severe general medical (e.g., tumors, transplants, serious accidents), neurological, or psychiatric comorbidity, substance abuse or alcohol dependence, traumatic brain injuries with loss of consciousness, or unclear or unverified previous psychiatric history. Any comparator (e.g., other psychotropic drug, sham or placebo treatment, psychotherapy, other active interventions, treatment as usual) was considered eligible, where applicable. We did not consider comedications as a potential exclusion criterion, aiming to describe a clinical framework as close as possible to everyday real clinical practice.

### 2.3. Outcome Measures

We settled as the primary outcome the evaluation of the risk of hip and/or vertebral fracture related to concomitant antidepressant use. The secondary outcome was the evaluation of any specific demographic or clinical feature related to the increased fracture risk. The absolute frequency or estimated increased fracture risk was considered potentially inclusive.

### 2.4. Data Extraction

Identified studies were independently screened and analyzed for eligibility by two authors (MM and GS) in a two-step double-blinded screening process; a first screening was performed based on the title and abstract, while full texts were read in full in case of inclusion in the second phase. At both stages, disagreements by reviewers were resolved by consensus or involvement of a third author (RdF) according to an already used methodology [15,16].

Article data were extracted by the same double-blind process by the same two authors through an ad-hoc developed data extraction spreadsheet developed after a first piloted extraction of 10 randomly selected papers and modified accordingly. Data extracted included sample sizes, demographic characteristics, fracture risk data, illness duration, types of disease, and treatment duration. Before data entry, values were converted to the same unit for each parameter and weighted means for covariates were computed based on means of subgroups.

### 2.5. Quality Assessment

The two reviewers (MM and GS) used the modified version of the Newcastle–Ottawa scale for cross-sectional studies as a quality assessment approach to independently rate the quality of the evidence included in this review [17]. Disagreements between reviewers were resolved by consensus or involvement of a third author (RdF).

#### Statistical Analysis

All data were collected and reported with one-decimal accuracy. The weighted mean, standard deviation, and range were noted for the continuous variables, as well as counts for the categorical variables. When the standard deviations were not directly provided, they were calculated with the equation [(max range−min range)/4] to allow for statistical aggregation [16]. We performed all statistical analyses with the JASP open-source software (JASP, version 0.16.1.0, University of Amsterdam, The Netherlands). We conducted a publication bias and adjusted meta-analysis applying a Robust Bayesian Meta-Analysis (RoBMA) method. We used a RoBMA with Competing Publication Bias Adjustment Methods [18] given the expected and probable results heterogeneity due to different patient populations, treatment interventions, comparators, and the innately considerable variability of the outcome variables and their detection. Indeed, this statistical method allows to correct for publication bias to give more weight to models that are better supported by data for the effect size (ES) or odd ratio (OR) estimation [18]. Finally, we calculated the final pooled ES using the single studies’ OR and their confidence intervals (CI) based on the sample size.

## 3. Results

**Figure 1:**
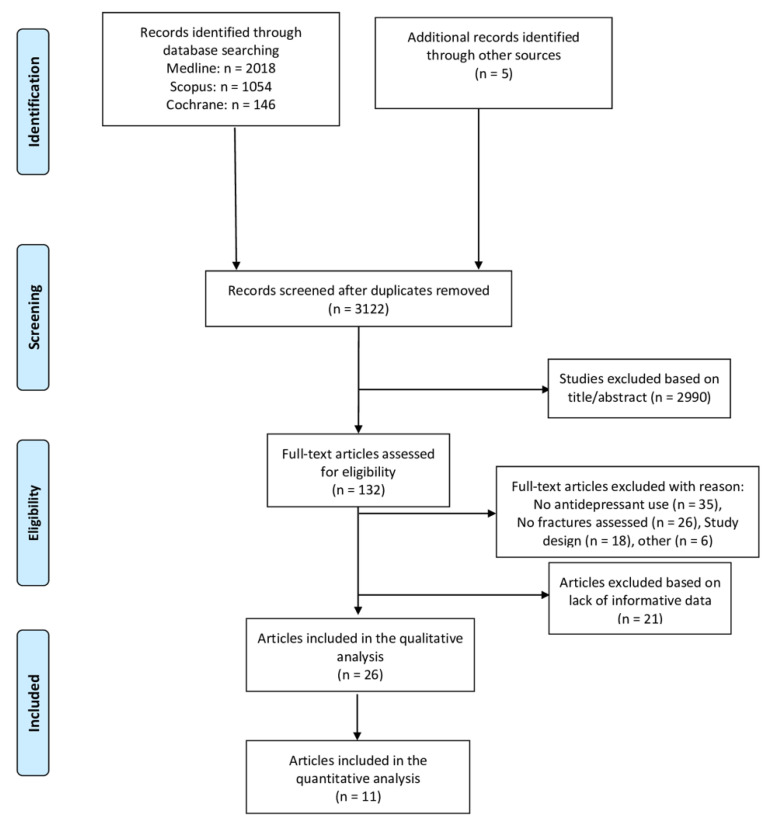
PRISMA Flow-chart.

After first conducting a search of the libraries and authors, and after removing duplicates, we found a total of 3122 articles to screen, of which 2990 items were excluded in the title/abstract phase because they did not fulfill the inclusion criteria or did not match the research topic. Afterwards, the full texts of the remaining 132 articles were carefully read and reviewed. In detail, 21 articles were excluded because of lack of informative data, while, of the remaining 111, 35 were removed because they did not involve antidepressants, 26 because they did not assess fracture risk, 18 due to study design (i.e., they were editorials, letters to editors, reviews, meta-analyses, or case reports), and, finally, 6 for other reasons. Thereafter, 26 papers were included in the qualitative analysis and 11 in the quantitative analysis based on the quality and quantity of the data reported (Figure 1).

### 3.1. Characteristics of Included Studies

The studies were conducted in several countries, including Australia, Canada, Finland, France, Israel, The Netherlands, Norway, Republic of Korea, Sweden, Taiwan, the United Kingdom, the United States of America, and have been published between 1998 and 2021. Briefly, the 26 studies included in this analysis contained data from 15,209,542 adult and elderly participants affected by major depressive disorders. Considering the 26 included studies, 25 evaluated SSRIs treatment, 16 provided data about TCA, 5 involved the use of SNRIs, and almost all of them compared several antidepressants in different arms. Nevertheless, we did not identify any other study using other types of antidepressants such as vortioxetine or esketamine. The studies enrolled only adult and elderly patients, ranging from young adults (18–35) to adults (36–64), and elderly (over 65). The gender ratio has only been presented in a few number of studies, and it tends to be unbalanced in favor of women. Fracture risk was reported as total number between groups or odd/hazard ratio with differences in values. The follow-up period reported in the included studies ranges from a minimum of 6 months to a maximum of 120 months, with an average of 51 months. The main characteristics and findings of included studies and their participants are reported in Table S1 in the Supplementary Materials [19,20,21,22,23,24,25,26,27,28,29,30,31,32,33,34,35,36,37,38,39,40,41,42,43,44].

### 3.2. Studies Quality Assessment

All studies included in the current meta-analysis were classified as high quality according to the modified version of the Newcastle–Ottawa scale (Table S2 Supplementary Materials) [19,20,21,22,23,24,25,26,27,28,29,30,31,32,33,34,35,36,37,38,39,40,41,42,43,44].

### 3.3. Analysis of Pooled Effect

After extracting the studies’ data regarding study design, population, setting, antidepressant category and dosage, comparators, physical and psychiatric comorbidities, fracture risk and site, outcomes, follow-up duration, and evaluation method, we found 11 studies with enough qualitative and quantitative information that were comparable in terms of characteristics to perform a meta-analysis [24,25,27,30,31,32,33,34,37,41,42].

**Figure 2:**
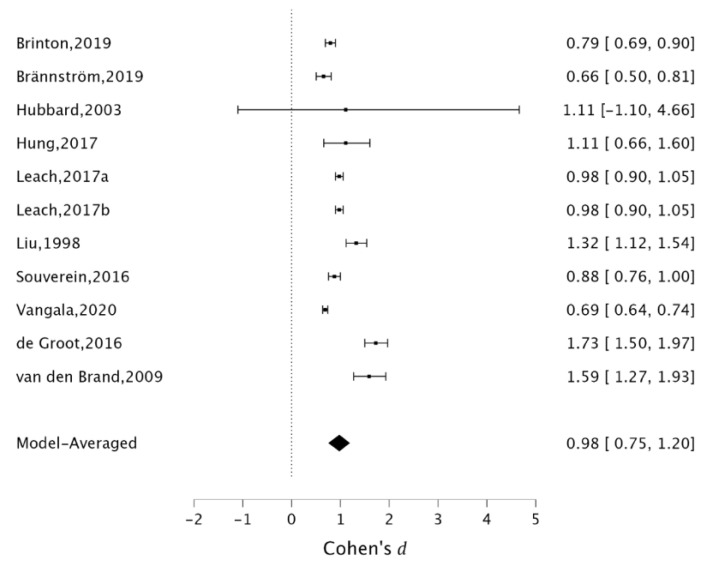
Analysis of pooled effect of SSRIs on increased hip risk fracture. The data was extracted from the following references [24,25,27,30,31,32,33,34,37,41,42].

Ten out of the eleven included studies had a significant single OR with CI and used SSRIs, with a minimum estimated effect of 0.66 (95% CI = 0.50, 0.81) [24], and a maximum estimated effect of 1.73 (95% CI = 1.50, 1.97) [27]. We found a significant positive effect of SSRIs on increased hip fracture risk with a mean effect of 0.98 (95% CI = 0.75, 1.20) (Figure 2).

Only three studies reported data on vertebral risk fracture and the large data heterogeneity of the included studies prevented us from conducting a comprehensive quantitative analysis between them or a specific sub-analysis regarding vertebral fracture.

## 4. Discussion

The widespread use of antidepressants worldwide and the concomitant high incidence of hip and vertebral fractures make the coupling between these two events increasingly frequent and, consequently, their causal connection plausible. Therefore, in this systematic review with meta-analysis, we aimed to explore the role of antidepressant treatment in increasing hip and vertebral fracture risk. In the current study, 26 studies were included in the qualitative analysis about vertebral and hip fracture risk related to all antidepressants, and 11 studies were considered in the quantitative synthesis focusing on SSRIs. A cumulative pooled effect of 0.98 (95% CI = 0.75, 1.20) in higher risk of hip fractures during SSRIs treatment for all causes was found.

The correlation between psychotropic drugs and both reduced BMD and increased risk of fractures has aroused considerable interest in recent years [21,45,46]. In fact, if on the one hand the risk of falls secondary to the use of benzodiazepines and hypnotics is well-known [47,48], then, more recently, other categories of psychiatric drugs have also been more intensely investigated [45,48]. Indeed, of particular interest is the correlation existing between increased risk of fracture and antidepressant prescription, which has been already largely investigated in literature [9,49]. However, the biological reasons of this observed connection are not clearly understood, since even depression itself was previously related to increased fracture risk in the general population [50,51]. On the other hand, more studies are needed to further explore the ambivalent role of antidepressants on both modulating osteoclastic activity and BMD balance, and their indirect outcome of their adverse drug reactions [52,53]. At the same time, it would be desirable to implement studies considering patients’ adherence and compliance to antidepressant treatment, in order to understand how much of their use really influences the outcome [54].

Vertebral, hip, and other sites fractures were already strongly related to specific psychiatric conditions, such as major depressive disorder and anorexia nervosa and even schizophrenia, especially in female populations with long life history of disease [55,56,57]. However, these data may be biased by the implicit gender-related higher risk of osteoporosis, iatrogenic hyperprolactinemia, and unhealthy lifestyle behaviors due to severe psychiatric diseases, thus limiting the generalization of the results to larger populations [23,58,59].

Our results found a significant positive correlation between antidepressants use and increased fracture risk, and this finding stands in line with some similar previous research studies [7,8,60,61,62] where several psychotropic medications were suspected to be culprit drugs for reduced BMD and increased fracture risk [63,64,65]. Another interesting aspect emerging from our results is the major SSRIs prescription compared to other antidepressants categories, including old TCA and new multimodal antidepressants. However, this finding could be confounding when interpreted in the light of a higher fracture risk associated with the SSRIs antidepressants class. It should be considered that currently SSRIs are the most commonly prescribed antidepressant category worldwide [66,67], because of their favorable cost/benefit ratio, availability, and easy handling [68], so that they also become the first line treatment for major depressive and anxiety disorder in most international guidelines [69,70].

The studies included in this systematic review identified a relatively long average follow-up, over 4 years, emphasizing that patients were treated for a medium-long amount of time, as well as that data about fracture risk should be sought after an appropriate time. This is not surprising considering that SSRIs are typically prescribed over a long period, and other research confirmed the same finding, with a higher absolute fracture risk related to longer use [63], while early fracture incidence could be secondary to possible quick adverse reactions during tapering, such as orthostatic hypotension, dizziness, falling, and tachyphylaxis [71,72,73].

One SSRI, paroxetine, has also been approved by the US Food and Drug Administration for the treatment of vasomotor menopausal symptoms, including hot flashes and night sweats that occur during the menopausal transition [74]. In this light, Sheu et al. found a higher risk of fractures in middle-aged women without mental disorders treated with SSRIs, an effect sustained over time, suggesting that shorter duration of treatment may decrease fracture risk [35].

Antidepressants are also widely used among patients with Alzheimer’s disease; Torvinen-Kiiskinen et al. reported that antidepressant use, including SSRIs, mirtazapine, and SNRIs, was associated with an increased risk of hip fracture among persons with and without Alzheimer’s disease [39]. The authors also found that the risk was not due to use of other fall-risk increasing psychotropic drugs, such as opioids and benzodiazepines.

The results of the current study should be interpreted in the light of several limitations and strengths, which we acknowledge. First, although the literature search was conducted without structural or drug category limitations, we did not identify studies using antidepressant categories aside from SSRIs, SNRIs, TCAs, and Noradrenergic and Specific Serotoninergic Antidepressants (NaSSA). However, it should not be interpreted as a results limitation, but rather as a suggestion for future studies that may also investigate the relationship between other antidepressants categories and the risk of fracture. Second, despite the large number of articles initially identified, the screening process led to the identification of only a small number for qualitative and quantitative evaluation, eventually leading to a meta-analysis of 11 papers. This problem was already identified by similar research [63] and also arose as a result of the high variability and heterogeneity of the collected data that did not allow for further generalization and aggregation of the data.

In addition, the heterogeneity of the studies included in the quantitative analysis and the poor identification of overlapping characteristics among the populations studied did not allow us to perform specific analyses on the influence of demographic factors (e.g., gender or age) or clinical factors (e.g., age of onset, length of disease) on the risk of fracture, so they continue to be relevant factors to explore in future studies. Finally, the lack of an adequate level of detail in the reported data and the methodological differences of the included studies prevent us from drawing firm conclusions, but rather help to define a risk trend without identifying a clear biological mechanism. However, we conducted an unrestricted literature review with respect to target population (e.g., psychiatric disease), intervention (e.g., antidepressant characteristics), comparator, or outcome to obtain results that were as naturalistic as possible, excluding only serious general medical comorbidities that could affect fracture outcomes.

## 5. Conclusions

This meta-analysis reveals that the use of SSRIs increases the risk of fractures. Not only are fragile categories, such as patients with major medical comorbidities, elderly, and postmenopausal women, subject to this observation, but also potentially anyone taking antidepressants, especially for a long time. A better understanding of the safety and tolerability profile of antidepressants should not discourage their use, but may increase their awareness in clinicians, providing a specific prescription to optimize the potential of antidepressants and reduce their risks.

## Data Availability

The data presented in this study are available on reasonable request from the corresponding author.

## Supplementary Materials

The following supporting information can be downloaded at: https://www.mdpi.com/article/10.3390/healthcare10050803/s1, Table S1: Characteristics of included studies providing the fracture number (n. 26), Table S2: Quality assessment of included studies according to the Modified Newcastle-Ottawa scale.
