# Supplementary material for: Antidepressants and Vertebral and Hip Risk Fracture: An Updated Systematic Review and Meta-Analysis
Source: Healthcare (Basel). 2022 Apr 26;10(5):803. doi: 10.3390/healthcare10050803 (PMC9140335; doi:10.3390/healthcare10050803)
Supplement: Supplementary file 1 [file healthcare-10-00803-s001.zip › healthcare-1665512-supplementary.pdf]

Supplementary Table S1. Characteristics of included studies providing the fracture number (n. 26).

Authors, year	Country	Study design	Antidepressant	Sample size at follow-up	Age of patients (mean ± SD)	Age of controls (mean ± SD)	Gender (M:F)	Fracture site	Fracture number main group	Fracture number control group	OR	CI 95%	Follow-up (months)
Aizenberg, 2015 [19]	Israel	Retrospective case-control study	SSRI<br>SNRI<br>TCA	372	84 ± 6,5	83 ± 5	57:128	Hip	35	35	N/A	N/A	N/A
Ali, 2016 [20]	Netherlands	Cohort study (Moondriaan cohort)	SSRI	169.948	47 ± 15	50 ± 16	N/A	Hip	35	47	N/A	N/A	N/A
		Cohort study (BIFAP cohort)		2.332.487	54 ± 17	51 ± 16			756	763			
Bakken, 2013 [21]	Norway	Prospective cohort study	SSRI	13.301	72	N/A	N/A	Hip	364	N/A	N/A	1,3-1,5	12
			TCA					Hip	3.272	N/A	N/A	1,7-1,8	
			Others						1.119			1,5-1,7	
Bali, 2016 [22]	USA	Retrospective cohort study	Paroxetine	4.620	65-85	N/A	N/A	Hip	213	N/A	0,35	0,91-1,32	N/A
			Other SSRI						217				
Bolton, 2017 [23]	Canada	Cohort study	SSRI	68.730	64 ± 11	64 ± 11	994:10.944	Hip	354	N/A	N/A	1,18-1,85	N/A
			TCA									0,96-1,51	
			Others									0,71-1,58	
Brännström, 2019 [24]	Sweden	Cohort study	TCA	408.144	80 ± 7	N/A	150.658:257.486	Hip	9.762	N/A	0,99	0,91-1,08	65
			Citalopram								0,91	0,81 – 1,01	
			Mirtazapine								0,94	0,75 – 1,18	
			Amitriptyline								1,18	0,79 - 1,76	
			Sertraline										
			Others										
Brinton, 2019 [25]	USA	Retrospective Cohort study	SSRI	11.349.632	85 ± 5	82 ± 7	46:118	Hip	N/A	N/A	1,57	1,46 – 1,68	120
Cumming, 1993 [26]	Australia	Case- control study	SSRI<br>SNRI<br>TCA	416	65 - 100	N/A	35:174	Hip	23	14	1,33	0,65 - 2,76	N/A
	Netherlands		SSRI	9.682	77 ± 13	N/A	2.074:7.608	Hip	2.155	N/A	3,13	2,77 – 3,52	79

De Groot, 2016 [27]		Case-crossover (UK population)	TCA						1.469		1,93	1,68 – 2,21	
		Case-crossover (Dutch Mondriaan population)	SSRI	277	79 ± 14		60:217		91		4,40	2,37 – 8,17	66
			TCA						28		1,42	0,62 – 3,22	
French, 2005 [28]	USA	Cross-sectional retrospective study	SSRI TCA	2.212	N/A	N/A	N/A	Hip	486	N/A	N/A	N/A	N/A
Gagne, 2011 [29]	USA	Cohort study	SSRI	2.711	77 ± 10	N/A	N/A	Hip	170	N/A	N/A	N/A	24
			Atypical antidepressant		77 ± 12				164				
			Secondary amine tricyclics		76 ± 10				144				
			Tertiary amine tricyclics		74 ± 12				155				
Hubbard, 2003 [30]	UK	Case-control study	Amitriptyline	46.230	N/A	N/A	N/A	Hip	29	13	3,73	1.88-7.42	N/A
			Dothiepin						22	13	2,72	1.29-5.73	
			lofepramine						22	4	9,96	3.24-30.59	
			Fluoxetine						16	3	8,59	2.42-30.48	
			Paroxetine						8	5	2,01	0.61-6.62	
Hung, 2017 [31]	Taiwan	Case-control study	SSRI	8.842	65-85	78,8	3669:6113	Hip	194	4289	3,85	2,13 - 6,94	6
Leach, 2017 [32]	Australia	Case-control study	Mirtazapine	44.138	88	88	3236:5592	Hip	375	1.026	1,27	1,12 – 1,44	N/A
			TCA						434	1.126	1,38	1,23 – 1,55	
			SSRI						1.232	2.598	1,77	1,64 – 1,91	
			SNRI						256	603	1,51	1,29 – 1,75	
Leach, 2017 [33]	Australia	Case-control study	SSRI	44.138	88	88	3236:5592	Hip	1.232	2.598	1,77	1,64 – 1,91	N/A
	France		SSRI	1.688	90 ± 6		N/A	Hip	891		2,4	2,0 – 2,7	N/A

Liu, 1998 [34]		Case-control study	Secondary amine tricyclics	673					400		2,2	1,8 – 2,8	
			Tertiary amine tricyclics	3.477					1.715		1,5	1,3 – 1,7	
Sheu, 2015 [35]	USA	Cohort study	SSRI	373.325	74 ± 8	74 ± 8	N/A	Hip	94	639	N/A	N/A	N/A
								Vertebral	299				
Shiri-Sharvit, 2005 [36]	Israel	Retrospective parallel group study	Carbamazepine<br>Tetracyclics<br>Trazodone<br>TCA<br>SSRI<br>SNRI	210	85 ± 11	81 ± 7	N/A	Hip	21	67	N/A	N/A	N/A
Souverein, 2016 [37]	Netherlands	Cohort and case control study (BIFAP)	SSRI	7.662	78 ± 11	78 ± 11	N/A	Hip	735	2.503	1.59	1.39-1.81	N/A
			TCA						93	368	1.37	1.06-1.66	
		Cohort and case control study (Mondriaan)	SSRI	384	75 ± 16	74 ± 15			33	79	3.03	1.59-5.80	
			TCA						11	45	1.68	0.77-3.68	
		Cohort and case control study (THIN)	SSRI	18.773	78 ± 13	78 ± 13			1.089	2.973	2.06	1.88-2.26	
			TCA						597	2.267	1.45	1.30-1.62	
Spangler, 2008 [38]	USA	Prospective cohort study	SSRI	93.675	N/A	N/A	N/A	Hip	1.132	N/A	N/A	1,14 – 1,63	48
								Vertebral	1.607				60
Torvinen-Kiiskinen, 2017 [39]	Finland	Retrospective cohort study	SSRI	151.473	80	80	6680:14650	Hip	391	N/A	N/A	N/A	48
			MIRTAZAPINE						172				
			SNRI						25				
Van de Ven, 2018 [40]	Netherlands	Cohort study	Antidepressant	1.766	75 ± 5	N/A	484:1282	Hip	282	N/A	N/A	14,3 – 17,9	12
Van den Brand, 2009 [41]	Netherlands	Case–control study	SSRI	33.104	70 ± 13	N/A	62:210	Hip	200	287	2,88	2,40 – 3,46	N/A
			TCA						172	323	2.22	1,84 – 2,68	
Vangala, 2020 [42]	USA	Case-control study	SSRI	4.912	72	62	N/A	Hip	N/A	N/A	1.26	1,12 – 1,41	69
Watt, 2018 [43]	Canada	Retrospective cohort study	Trazodone<br>Atypical antidepressant	9.463	85 ± 7	85 ± 7	1994:4594	Hip	4	5	N/A	0,53 – 1,32	N/A
Yang, 2021 [44]	Republic of Korea	Self-controlled case-series analysis	TCA<br>SSRI<br>Other	3.077	45 ± 11	N/A	706:2314	Hip	462	N/A	N/A	N/A	N/A
								Vertebral	1.728				

SD: standard deviation; M: male, F: female; N/A: Not available; OR: odds ratio; CI: confidence interval; BIFAP: Base de datos para la Investigación Farmacoepidemiológica en Atención Primaria; THIN: The Health Improvement Network.

Supplementary Table S2. Quality assessment of included studies according to the Modified Newcastle-Ottawa scale.

Study Author (year)	Criteria								Total	Quality
	1	2	3	4	5	6	7	8		
**Aizenberg et al. (2015) [19]**	1	1	1	1	1	2	1	1	9	High
**Ali et al. (2016) [20]**	0	0	1	1	1	2	1	1	7	High
**Bakken et al. (2013) [21]**	1	0	1	1	1	1	1	1	7	High
**Bali et al. (2016) [22]**	1	0	1	1	1	2	1	1	8	High
**Bolton et al. (2017) [23]**	0	0	1	1	1	2	1	1	7	High
**Brännström et al. (2019) [24]**	1	0	1	1	1	2	1	1	8	High
**Brinton et al. (2019) [25]**	0	0	1	1	1	2	1	1	7	High
**Cumming et al. (1993) [26]**	1	1	1	1	1	2	1	1	9	High
**De Groot et al. (2016) [27]**	1	0	1	1	1	1	1	1	7	High
**French et al. (2005) [28]**	0	0	1	1	1	2	1	1	7	High

**Gagne et al. (2011) [29]**	1	1	0	1	1	1	1	1	7	High
**Hubbard et al. (2003) [30]**	0	0	1	1	1	2	1	1	7	High
**Hung et al. (2017) [31]**	1	1	1	1	1	1	1	1	8	High
**Leach et al. (2017) [32]**	1	0	1	1	1	2	1	1	8	High
**Leach et al. (2017) [33]**	1	0	1	1	1	2	1	1	8	High
**Liu et al. (1998) [34]**	1	0	1	1	1	2	1	1	8	High
**Sheu et al. (2015) [35]**	1	1	1	1	1	2	1	1	9	High
**Shiri-Sharvit et al. (2005) [36]**	1	1	1	1	1	1	1	1	8	High
**Souverein et al.(2016) [37]**	1	1	1	1	1	1	1	1	8	High
**Spangler et al. (2008) [38]**	1	1	1	1	1	2	1	1	9	High
**Torvinen-Kiiskinen et al. (2017) [39]**	1	1	1	1	1	2	1	1	9	High
**Van de Ven et al. (2018) [40]**	0	0	1	1	1	2	1	1	7	High

**Van den Brand et al. (2009) [41]**	1	1	1	1	1	1	1	1	8	High
**Vangala et al. (2020) [42]**	0	0	1	1	1	2	1	1	7	High
**Watt et al. (2018) [43]**	1	1	1	1	1	1	1	1	8	High
**Yang et al. (2021) [44]**	1	0	1	1	1	2	1	1	8	High

Based on the total score, quality was classified as “low” (0-3), “moderate” (4-6) and “high” (7-9). Criterion number (in bold): 1, representativeness of the exposed cohort; 2, selection of the nonexposed cohort; 3, ascertainment of exposure; 4, demonstration that outcome of interest was not present at start of study; 5, comparability of cohorts on the basis of the design or analysis; 6, assessment of outcome; 7, was follow-up long enough for outcomes to occur?; 8, adequacy of follow up of cohorts. Each study was awarded a maximum of one or two points for each numbered item within categories, based on the Modified Newcastle-Ottawa scale rules
